# Bone substitute in distal radius osteotomy: a prospective randomized double-blinded study on micromotion and outcomes in 42 patients up to one year

**DOI:** 10.1007/s00402-025-05800-4

**Published:** 2025-03-10

**Authors:** Vasileios Angelomenos, Anders Björkman, Bita Shareghi, Ingrid Andreasson, Michael Ullman

**Affiliations:** 1 University of Gothenburg, Gothenburg, Sweden; 2 Sahlgrenska University Hospital, Gothenburg, Sweden

**Keywords:** Wrist fractures, Osteotomy, Radiostereometric analysis, Bone substitutes, Radius fractures, Malunion

## Abstract

**Introduction:**

Symptomatic malunion following a distal radius fracture (DRF) is commonly treated with a corrective osteotomy. Such osteotomy is traditionally fixed with a palmar plate in combination with autogenous bone graft in the osteotomy gap. However, bone grafting prolongs surgery and may result in comorbidity. Recent studies suggest that filling the osteotomy gap may not be necessary. An alternative is to fill the gap with an injectable bone substitute (IBS). However, there is limited data on inter-fragmentary micromotion in osteotomies utilizing IBS. This study aims to assess micromotion and outcomes in patients undergoing corrective osteotomy for malunited DRFs, comparing outcomes between those treated with and without IBS.

**Materials and methods:**

Patients undergoing distal radius osteotomy for symptomatic malunion were randomized to either an IBS (hydroxyapatite, HA) group or control group (where the osteotomy gap was left unfilled) in combination with palmar plate fixation. Radiostereometric Analysis (RSA) markers were placed in the radius, and RSA assessments were conducted immediately postoperatively and at 6 weeks, 3, 6, and 12 months. The primary outcome of the study was dorsal/palmar tilt, while the secondary outcomes were radial shortening, radial inclination, radial shift, as well as clinical and Patient Reported Outcomes (PROMs).

**Results:**

42 patients (24 control, 18 HA-group) were included in the analysis. Significantly less micromotions were noted in the HA-group at multiple follow-ups (*p* ≤ 0.05) in X-axis rotation and Y-axis translation, however they were in the subclinical scale. Both groups showed functional improvements over time, but there were no differences between the groups in clinical outcomes or PROMs.

**Conclusions:**

Hydroxyapatite bone substitute does not offer additional benefits in terms of stability or outcomes in extra-articular corrective osteotomy for malunited DRFs when a palmar plate is used for fixation and palmar cortical bone contact is maintained.

## Introduction

Distal radius fracture (DRF) is the most common fracture type, accounting for 10–12% of all fractures [1, 2]. The majority of patients demonstrate a positive response to treatment, with approximately 25% experiencing persistent symptoms, including functional impairments and pain [3]. A primary cause of such persisting symptoms is malunion of the DRF. Malunion is the result of axial shortening and dorsal angulation of the radius and results in ulnocarpal impaction and incongruity in the distal radioulnar joint, potentially leading to wrist instability, diminished wrist and forearm mobility, radiocarpal arthritis and pain [3]. Symptomatic malunited DRFs are typically managed with corrective osteotomy, aimed at restoring or improving anatomical alignment [3–5]. During this procedure, the distal radius is surgically osteotomized, and the distal segment including the joint surface is repositioned to a more anatomical position, then stabilized with a palmar plate. Reduction typically involves extending the radius, which results in the formation of an osteotomy gap. This gap can be filled with autologous bone graft, injectable bone substitute, or occasionally left unfilled [1, 2]. The use of autogenous iliac crest cancellous or corticocancellous bone grafts have long been the standard approach for distal radius osteotomies. These grafts offer several advantages, including the ability to fill the gap, enhancing stability, and providing growth factors as well as a scaffold for rapid bone formation and healing [6]. However, the graft is typically harvested from the iliac crest, which prolongs surgery. One study found that 73% of procedures were delayed by 20 min or more due to this step [2]. Moreover, general anaesthesia is necessary to harvest iliac bone compared to regional anaesthesia if only the arm is being operated on. Additionally, graft harvesting often results in donor site morbidity [1, 7–9]. Recent studies suggest that after an open wedge osteotomy of the distal radius where palmar cortical contact is maintained, it may be unnecessary to fill the osteotomy gap [1, 10]. Some evidence supports leaving the gap unfilled also in cases when there is no bone contact between the proximal and distal segments [11]. This approach, however, may increase strain on the osteosynthesis, with a theoretical risk of hardware bending or loosening, as well as the potential for prolonged healing times [2]. An alternative to autologous bone grafting is the use of injectable bone substitutes (IBS), such as calcium phosphate-based materials. These substitutes are designed to enhance stability and promote healing compared to leaving the gap empty [12]. In vivo studies have shown that calcium phosphate substitutes undergo remodeling through processes like osteoclastic activity and vascular infiltration, while also providing a structural framework for osteoblasts to form new bone [13]. Several case series have assessed the effectiveness of different bone substitutes in maintaining palmar tilt and promoting bone union with most of them showing positive results with stable fixation, good healing and no donor morbidity [14–16]. However, some studies showed no added clinical benefits in terms of fixation and function [17]. Furthermore, some limitations exist with IBS, including concerns over the injection of synthetic substances and the high cost of these materials compared to autologous grafts. Interestingly, to our knowledge, no studies have yet directly compared micromotion of the distal radius osteotomy segment with and without the use of IBS.

Since its introduction by Selvik in 1974 [18], marker-based Radiostereometric Analysis (RSA) has been the gold standard in measuring micromotions of orthopaedic implants of the hip, knee and shoulder [19]. However, there are only a handful of studies utilizing RSA in analysis of micromotion in the wrist after injury or osteosynthesis due to a fracture or osteotomy of the wrist [16, 20, 21].

Our study was designed to evaluate the performance of injectable bone substitutes, as they are increasingly used in clinical practice to avoid donor site complications. The aim of this study was to investigate the interfragmentary micromotion and evaluate clinical outcomes and PROMs (Patient Reported Outcome Measures) in patients undergoing open-wedge corrective osteotomy and palmar plate fixation for malunited DRF, treated with or without IBS.

## Methods

Consecutive patients scheduled for an osteotomy of the distal radius due to an extra-articular malunion at the department of Orthopaedics in our hospital between 2014 and 2018 were screened for inclusion in the study. The study was approved by the local ethics authority. All patients received oral and written information about the study and gave informed consent to participate before the surgery.

The inclusion criteria were: age between 16 and 85 years, diagnosis of a symptomatic distal radius malunion (DRM) with a dorsal tilt of > 20° with or without one or more of the following parameters: radial inclination < 10°, radial shift > 5–10 mm or radial shortening > 5 mm, at least 3 months after conservatively treated extraarticular distal radius fracture and suitable for extra-articular distal radius osteotomy. Exclusion criteria were: cases which were deemed to need correction without the possibility to maintain any cortical contact, such as pronounced distal radius deformity that called for distraction of the radius and ulna variance > 5 mm, intraarticular step-off that would need an intraarticular osteotomy, radiological signs of osteoarthritis in the radiocarpal, midcarpal and DRU-joint, active drug abuse, compromised soft tissue conditions, severe soft tissue contractures, fixed carpal malalignment, and patients with significant comorbidities or with mental disorders inhibiting adherence to treatment and rehabilitation and inability to read or lack of language skill.

The patients were randomized, using a computer randomization software, on the day of the surgery to be operated with osteotomy and palmar plate fixation in combination with injection of a hydroxyapatite osteoconductive bone substitute, Hydroset^®^ (Stryker Corp, Kalamazoo, MI, USA) in the osteotomy gap or leaving the osteotomy gap unfilled. In all cases palmar cortical contact was maintained.

### Surgical technique

All participants were operated by the same senior consultant in orthopaedics. A longitudinal dorsal incision of 35–40 mm centered over Listers tubercle was made. The 3rd extensor compartment was released, and the 2nd and 4th extensor compartments were elevated subperiostealy to expose the dorsal aspect of the distal radius. The planned osteotomy site was indicated with a K-wire. Prior to performing the osteotomy, six to seven Tantalum markers™ (1.0 mm diameter; RSA Biomedical, Umeå, Sweden) were inserted into the proximal and distal bone segments using a specialized inserter (RSA^®^ Injector™, RSA Biomedical, Umeå, Sweden), following established RSA guidelines [22]. The osteotomy was performed via the dorsal incision with a power saw and the distal segment was positioned in the most anatomically correct position possible. A set of preformed plastic wedges with different angles where then used. The most appropriate wedge was selected according to the intraoperative radiograph and was inserted dorsally in the osteotomy gap and fixed with two K-wires, thus stabilizing the osteotomy. In doing so, care was taken to maintain a palmar cortical contact. In cases with significant relevant shortening of the radius in this cohort, it was still possible to reduce the distal radius while maintaining cortical contact in the palmar-ulnar corner of the radius.

**Fig. 1:**
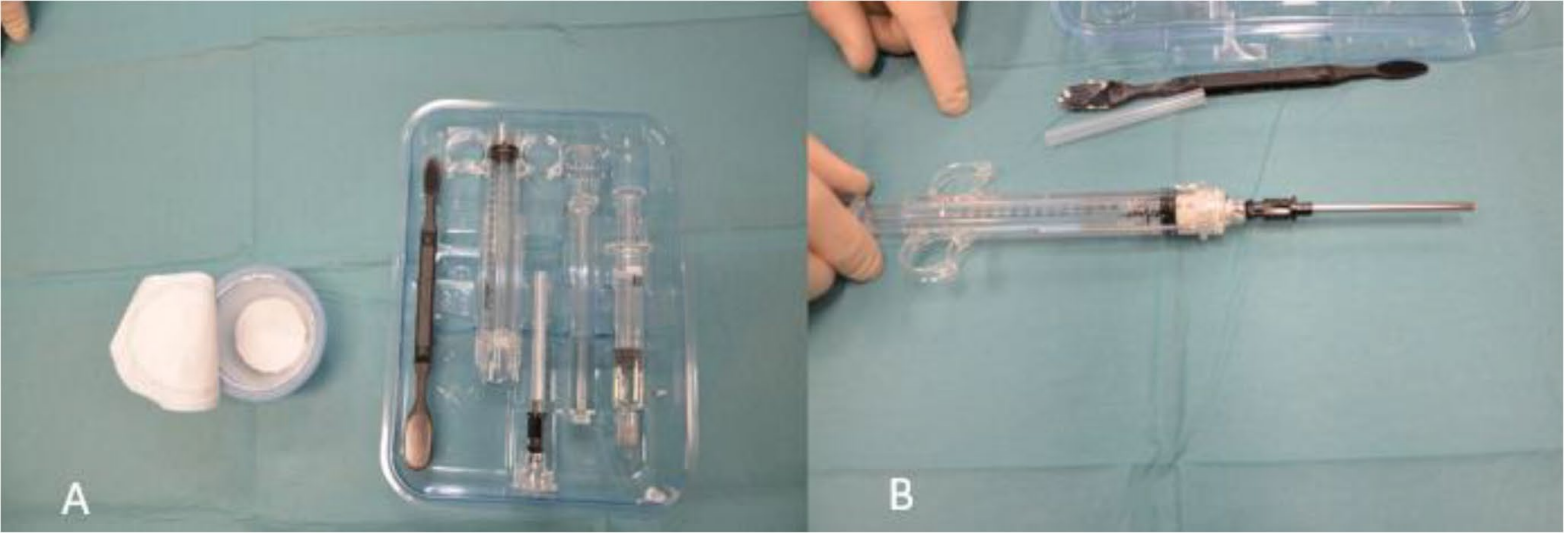
Picture of the injectable bone substitute set (Hydroset). The set includes a powder and liquid component and mixing instruments (**A**). After the mixing is complete the mixture is transferred to a special syringe to be injected in the osteotomy gap (**B**)

A modified Henry approach was then used to access the palmar distal radius. Thereafter, the osteotomy was stabilized with a palmar DiPhos R^®^ radiolucent plate (Lima Corporate, Udine, Italy), made of carbon fibers and PEEK (PolyEtherEtherKetone-polymer) fixed with titanium screws. The plate was manufactured with embedded tantalum markers of 0.8 mm diameter, to make it visible on the radiographs. As the beads are smaller than the intra-osseous markers, they can be distinguished on RSA analysis. When the plate was fixated, care was taken to secure that some palmar cortical contact remained. Following plate fixation, the wedge was removed, and the osteotomy gap was filled with hydroxyapatite bone substitute (Hydroset^®^, Stryker Corp, Kalamazoo, MI, USA) (HA-group) (Fig. 1) or left unfilled (control group).

Following plate fixation, the palmar and dorsal incisions were closed in layers with resorbable sutures, and the wrist was immobilised in a Softcast^®^ for two weeks.

### Rehabilitation

Between four to seven days after surgery, patients visited a hand therapist and were instructed to use the hand for light daily activities and to begin hourly exercises to reduce hand oedema. After two weeks, the cast was replaced with a brace (Wrist Lacer short 28571, Camp Scandinavia AB, Helsingborg, Sweden), and gentle wrist range-of-motion exercises were introduced four times daily as part of a home exercise program. All participants were also seen by therapist four weeks after surgery and after that visits at the hand therapist was personalized based on recovery of function.

### Follow up of clinical outcomes and PROMs

An occupational therapist, not involved in the clinical care of the patients, experienced in hand and wrist rehabilitation conducted all assessments. Both the patients and the therapist assessing functional outcomes were blinded to group allocation. Assessments were performed preoperatively and 12 months postoperatively. Regarding clinical outcomes, Range of Motion (ROM) of the wrist and grip strength were evaluated. Grip strength was measured using a Jamar hydraulic hand dynamometer (Performance Health, Warrenville, Illinois, USA), with the mean of three measurements recorded [23]. PROMs were evaluated using the Swedish versions of the Disability of the Arm, Shoulder and Hand questionnaire (Quick-DASH) [24], the Patient Rated Wrist Evaluation (PRWE) questionnaire [25], as well as a visual analogue scale for pain (VAS-pain) [26].

Quick-DASH scores range from 0 to 100, with lower scores indicating better function and less pain [24, 27]. The PRWE include five pain-related and ten function-related questions. Scores range from 0 (no pain, full function) to 100 (severe dysfunction and pain) [25, 28].

### Radiostereometric analysis (RSA)

All marker-based RSA measurements and analyses were conducted with UmRSA Digital Measure and Analysis software, version 7.0 (RSA Biomedical, Umeå, Sweden), which calculates rotation and translation along the three orthogonal axes (x, y, z) that correspond to the coronal, sagittal and transverse planes respectively, as shown in Fig. 3. RSA examinations were performed immediately post-surgery and at intervals of 6 weeks, 3, 6, and 12 months. This marker-based RSA method adheres to ISO standards (ISO16087:2013 [22], requiring that a minimum of either 25% of cases or 15 cases (whichever is larger) undergo duplicate examinations to ensure precision.

**Fig. 2:**
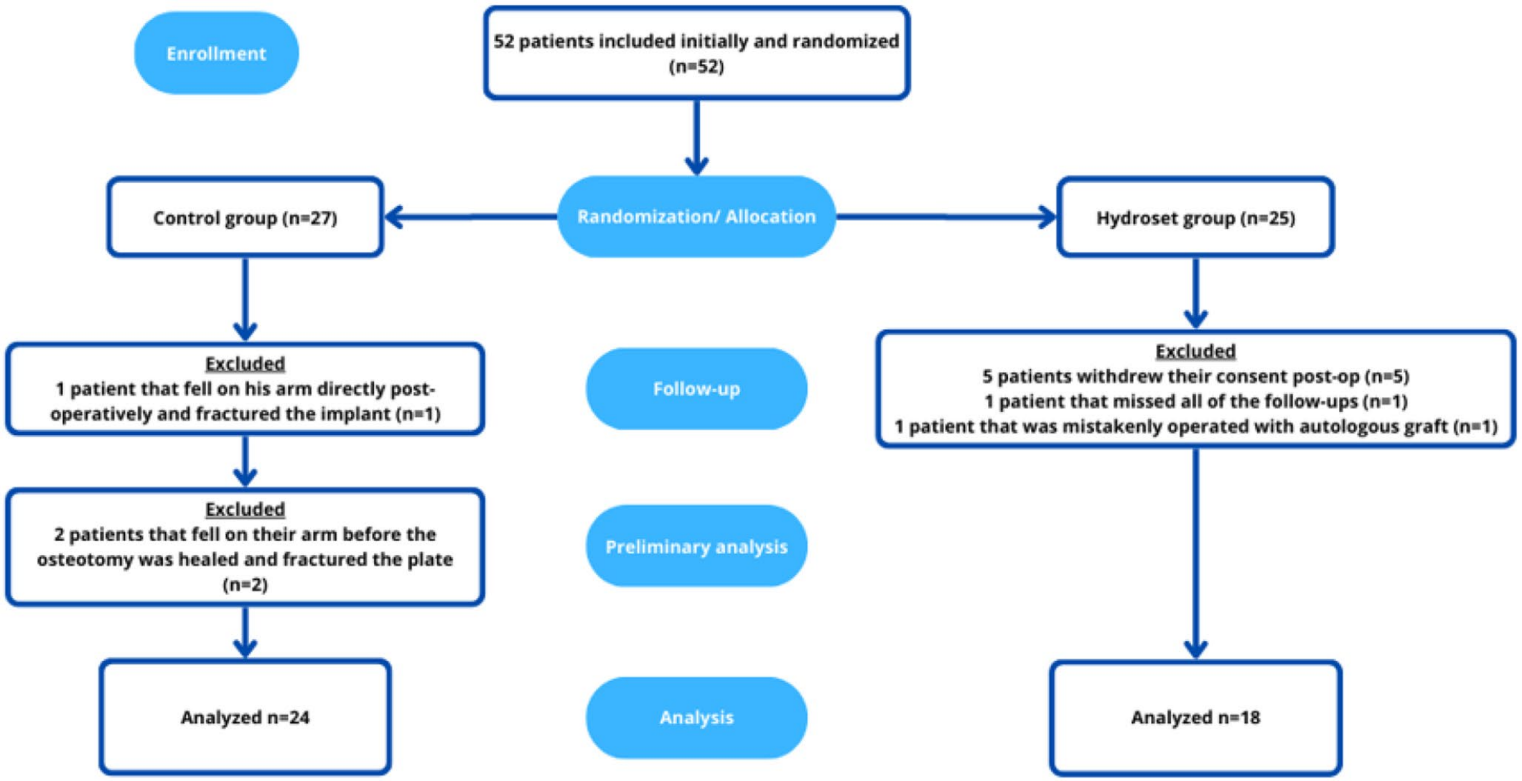
Flowchart showing patient inclusion workflow

To evaluate RSA precision, 19 double RSA measurements were conducted after clinical and radiological confirmation of osteotomy healing, recording any differences in translation and rotation. These double measurements, taken minutes apart, involved repositioning of the patient’s hand without modifying the RSA setup (the calibration cage and X-ray tubes remained unchanged) [29]. It was assumed that there was no actual movement of the plate, radius shaft, or distal radius segment between the two consecutive RSA examinations.

All imaging was performed using an Adora radiographic system (NRT-Nordisk Røntgen Teknik A/S, Hasselager, Denmark), applying a uniplanar technique with an RSA calibration cage positioned beneath the table (cage 77, RSA Biomedical, Umeå, Sweden). A single biomedicine scientist with extensive clinical and research expertise in RSA handled all analyses.

For larger bones and implants, a condition number (CN) limit of 120–150 is recommended [30]; however, in smaller bones like those of the wrist and hand, higher CNs are expected due to the limited bone area for marker placement [18, 27, 28]. In the RSA software, the mean error of rigid body fitting reflects the stability of bone markers, with an upper limit of 0.35 mm suggested [30]. Per ISO16087:2013 [22], when high CN values are accepted, it is essential to validate RSA measurement precision, as done in this study through double examinations. In the current study, stereoradiographs were evaluated only when 3 or more tantalum markers were visible in both distal and proximal osteotomy segments, with a CN ≤ 200 and a mean error of rigid body fitting  ≤ 0.35 mm [31, 32].

### Outcome measures

**Fig. 3:**
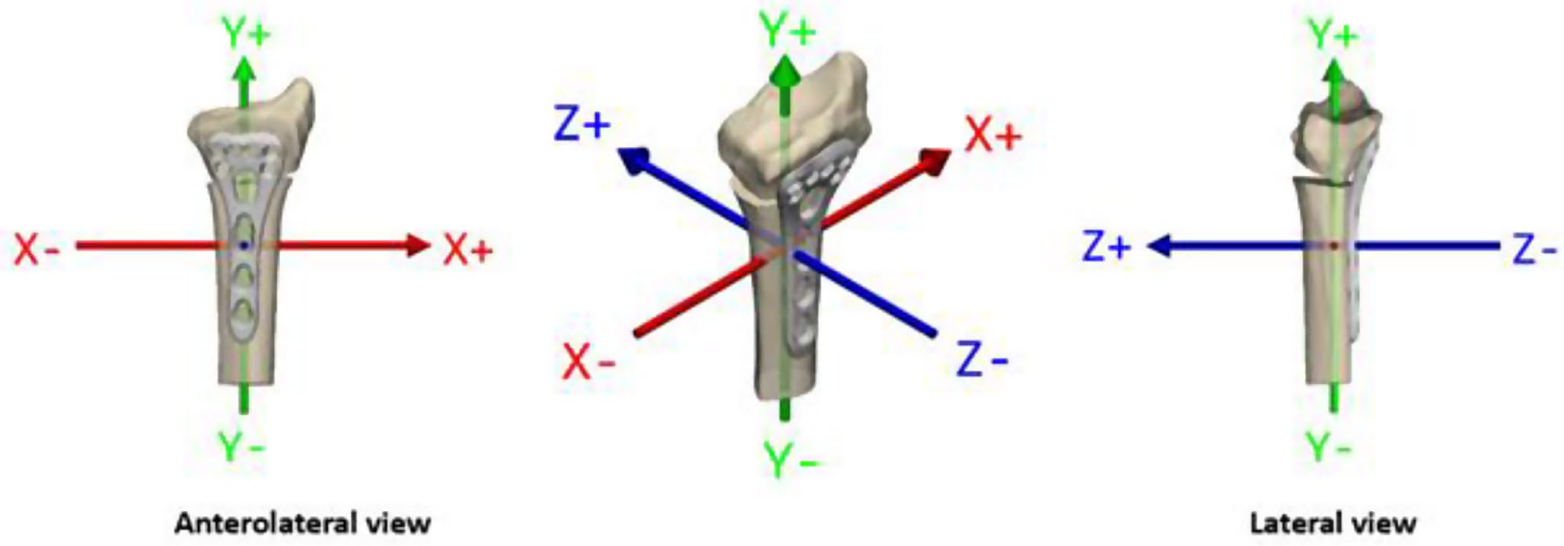
Axes of interest in distal radius displacement include the following: dorsal/palmar tilt corresponds to rotations around the X-axis, radial inclination to rotations around the Z-axis, axial compression to translations along the Y-axis, and radial shift to translations along the X-axis

The primary outcome measure was the dorsal/palmar tilt of the distal radius segment which equals to rotation around the X-axis. Secondary outcomes were: the axial translation of the distal radius osteotomy segment which equals to translation along the Y-axis, change in radial inclination which equals to rotations around the Z-axis and radial shift which equals to translation along the X-axis (Fig. 3). Additional secondary outcomes were the differences in ROM of the wrist, grip strength, and PROMs between the two patient groups.

### Coordinate system

A standard RSA coordinate system for the wrist was used in this study (Fig. 3). For translations on the X-axis (ulnar/radial) negative values correspond to ulnar translations, while in the radial direction to positive. Correspondingly, on the Y-axis (proximal/distal) translations in the proximal direction are negative, and in the distal direction positive and on the Z-axis (palmar/dorsal translations) palmar translations are negative, while dorsal are positive. For rotations on the X-axis (dorsal/palmar tilt) negative values indicate dorsal tilt, and positive values palmar tilt. For the Y-axis (internal/external), negative values represent internal rotation and positive values external rotation and for the Z-axis (increased/decreased radial inclination), increased radial inclination is represented by negative values, while decreased by positive.

### Radiation dose

The mean effective dose of marker-based RSA was estimated to be 0.02 µSv per scan. For comparison, one simple set of dental radiographs generates about 5–10 µSv [33].

## Statistics

### Power calculation

There is to our knowledge no predefined number of patients for marker-based RSA studies of distal radius osteotomies. Based on previous research on experimental and clinical wrist RSA studies [34, 35], as well as studies and guidelines for RSA studies of larger anatomical areas [30, 36] we estimated that 40 patients were needed in this study. A priori power analysis with a level of significance of α = 0.05 and power β = 0.8 with an effect size of d = 0.65, showed that a sample of at least 40 patients was needed. In order to compensate for dropouts and patient exclusion due to insufficient bone marker placement, which is a well-known issue in RSA studies [30], we decided to include 52 patients in this study.

### Distal radius migration

**Fig. 4:**
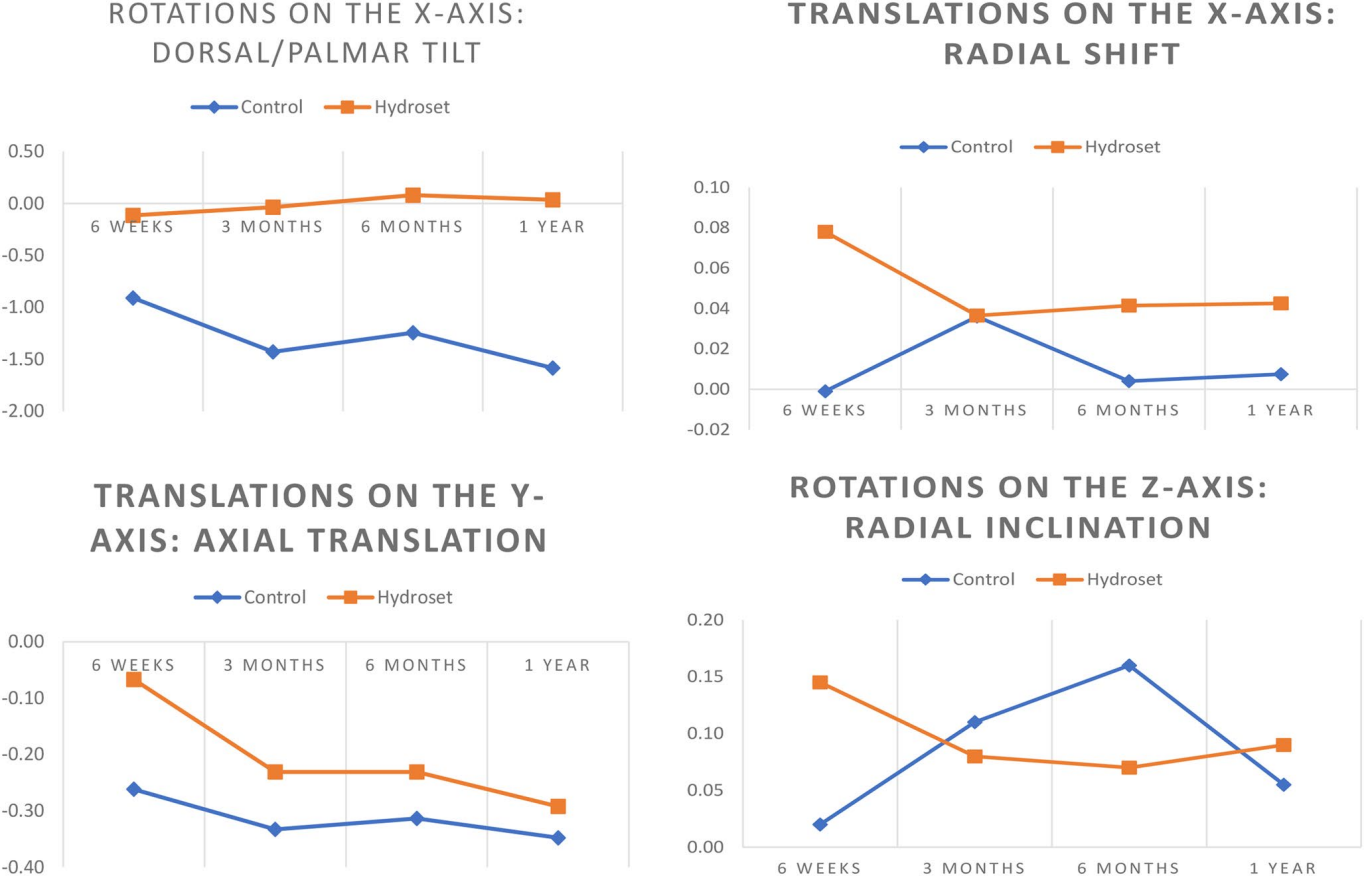
Graphical depiction of progress over time of the corresponding studied micromovements presented as median micromovement. The micromovements of the control group are presented as a blue line and those of the hydroset group as an orange line. Translations are presented in mm and rotations in degrees

All statistical analysis was performed using IBM SPSS ^®^ version 27.0.0 software. The level of significance was set to 0.05 for all tests (α = 0.05) and all performed tests were two-tailed. In order to ensure the quality of the reduction of the distal radius in both groups after the osteotomy, the postoperative palmar and radial inclination as well as ulnar variance was measured on plain postoperative radiographs and evaluated. The data was examined using a histogram and density curve and found to be normally distributed. The mean (SD) and range of the parameters above were calculated and compared for both groups using Student’s T-test. Descriptive statistics were used to calculate standard deviation (SD) of the differences between the double RSA examinations. The precision of the RSA measurements was defined as “the degree to which repeated measurements under unchanged conditions show the same results and it refers to random errors only” [37]. The formula used to calculate precision was defined as Precision = SD × t_(n− 1)_ [38], where (SD) corresponds to the standard deviation of the differences calculated between double RSA examinations multiplied by the critical value (t) found in the t-table adjusted for the number of observations minus 1 (n-1). The double marker-based RSA analysis was done when the osteotomy was deemed to be clinically and radiologically healed. Thus, it was assumed that no true motion of the plate or the distal radius segments occurred between the examinations in order to calculate precision [38]. The small sample size prompted an examination of normality of data using a histogram with a density curve. The data distribution was graphically assessed and found to be non-normal, thus the Mann-Whitney U test was used to assess any differences between groups. The median and interquartile range (IQR) of rotation around the X-axis, translation along the Y-axis, rotation around the Z-axis and translation along the X-axis, at the predefined time points were calculated using descriptive statistics, and furthermore, a graphical evaluation of the same measures was performed (Fig. 4).

### Clinical outcomes and proms

Data was assessed for normality using a histogram with density curve and was found to be non-normal. Thus, the Mann-Whitney U test was used to assess any differences in ROM of the wrist, grip strength, and PROMs between the two patient groups. Furthermore, ROM, grip strength, and PROMs were presented as median (IQR) preoperatively and at 1 year.

## Results

From 2014 to 2018, 52 patients, 12 men and 40 women, with a median age of 63 years (range 16 to 85 years) were included in the study. The randomization software that was used had some flaws and lacked the possibility of back-up. As one smartphone that was used for the randomisation process was lost, a minor imbalance between inclusions in the two the groups was created, thus, 25 patients were randomized to the HA-group and 27 to the control group instead for 26 patients in each group. However, from the HA-group, five patients did not wish to take part in the study post-operatively and withdrew their consent and one patient missed all the post-operative follow-ups. Moreover, one patient who was randomised to the HA-group, was operated by another surgeon who mistakenly used autologous bone graft. Additionally, one patient from the control group fell on his arm directly post-operatively on the day of the operation and fractured the newly implanted plate which led to a reoperation later. These eight patients were excluded from the analysis. In addition, in the preliminary statistical overview, two extreme outliers, both from the control group, were noted. After review of their medical journal, it was noted that both patients have had a traumatic fall against their arm between the 6- and 12-week follow-up postoperatively before the osteotomy was healed and as a result the plate that was used to stabilize the osteotomy had broken. These patents were also excluded from the analysis.

In summary, 42 patients were left for the definitive analysis, 24 patients in the control group and 18 in the HA-group (Fig. 2). Demographics of the patients are presented in Table 1. No statistical difference between groups in terms of gender or age distribution were noted.

**Table 1:** Demographics of patients eligible for the 1-year follow-up. HA-group refers to the hydroset group. M: male and F: female. Age is presented as median (range). P-values are also presented and no statistical difference between groups in terms of gender or age distribution were noted

Group	Number of participants	Gender Male/Female	Age
Control group	24	6/18	61 (24 to 68)
HA-group	18	4/14	63 (21 to 80)
P-values		**0.44**	**0.53**

### Distal radius segment migration

There was no difference in postoperative palmar and radial inclination, as well as ulnar variance between the groups (Table 2). The precision of the RSA method for the primary measure outcome (rotations around the x-axis) was 1.03**°.** The precision of the RSA method on the rest of the axes are summarized on Table 3. The median (IQR) rotation on the x-axis at 1 year was 0.04° (1.81°) for the HA-group and − 1.59° (3.03°) for the control group, while corresponding values on the z-axis were 0.09° (1.2°) and 0.06° (0.94°). The median (IQR) translation on the y-axis were-0.2 (-0.4) mm for the HA-group and − 0.35 (-0.32) mm for the control group, while corresponding values on the x-axis were 0.04 (0.53) mm and 0.01 (0.6) mm (Tables 4, 5, 6, 7). The graph on dorsal/palmar angulation (rotation on the X-axis) showed that the HA-group exhibits less loss of angulation in absolute numbers at all time points. The graph on axial compression (translation along the Y-axis) showed that the HA-group demonstrated less axial compression in absolute numbers at six weeks, that later over time converges to that of the control group at one year (Fig. 4). Furthermore, the HA-group showed significantly less rotation on the X-axis at all follow-ups (*p* = 0.017 at 6 weeks, *p* = 0.047 at 3 months, *p* = 0.049 at 6 months and *p* = 0.014 at 1 year), as well as less translation on the Y-axis at 6 weeks (*p* = 0.001) and 6 months (*p* = 0.043). For rotations around the Z-axis and translations on the X-axis no statistical differences between the groups were found at any time point (Tables 4, 5, 6 and 7).

**Table 2:** Postoperative palmar and radial inclination presented as degrees, as well as ulnar variance presented in mm for both groups. The mean (SD) and range are presented along with the corresponding P-values

Group	Palmar inclination		Radial inclination		Ulnar variance	
	Mean (SD)	Range	Mean (SD)	Range	Mean (SD)	Range
Control group	3.5 (4.2)	-4.9 to 11.9	23.6 (4.8)	14.0 to 29.2	0 (0.75)	-1.5 to + 1.5
HA-group	4.3 (3.4)	-2.5 to 11.1	22.2 (3.2)	15.8 to 28.6	0 (1)	-2 to + 2
P-values	**0.85**		**0.64**		**0.76**	

**Table 3:** Precision of marker-based RSA in measuring distal radius segment migration and rotation based on 19 double examinations. Precision is defined as the margin of error that is equal to standard deviation (SD) X critical value from the t-table adjusted for the number of observations minus 1. A 95% significance level was used

	RSA	
	SD	Precision
**Translations (mm)**		
X-axis (ulnar/radial)	0.14	0.29
Y-axis, (proximal/distal)	0.04	0.09
Z-axis, (palmar/dorsal translation)	0.21	0.44
**Rotations (degrees)**		
X-axis, (dorsal/palmar tilt)	0.49	1.03
Y-axis, (internal/external)	0.40	0.83
Z-axis, (increased/decreased radial inclination)	0.40	0.84

**Table 4:** Rotations on the X-axis. Palmar and dorsal Tilt of the distal radius segment presented as in degrees as median (IQR). Negative values correspond to dorsaltilt, while positive values to palmar tilt. P-values of the Mann-Whitney U test for the two groups is also presented

Group/ Time	6 weeks	3 months	6 months	1 year
Control	-0.91 (2.31)	-1.43 (2.14)	-1.25 (2.35)	-1.59 (3.03)
Hydroset	-0.12 (1.14)	-0.04 (2.37)	0.08 (1.59)	0.04 (1.81)
**P-values**	***0.017***	***0.047***	***0.049***	***0.014***

**Table 5:** Translations on the Y-axis. Axial (proximal/distal) translation of the distal radius segment presented in mm as median (IQR). Negative values correspond to proximal translation and positive values to distal translation. P-values of the Mann-Whitney U test for the two groups is also presented

Group/ Time	6 weeks	3 months	6 months	1 year
Control	-0.26 (-0.27)	-0.33 (-0.26)	-0.31 (-0.27)	-0.35 (-0.32)
Hydroset	-0.07 (-0.24)	-0.23 (-0.33)	-0.23 (-0.42)	-0.2 (-0.40)
**P-values**	***0.001***	0.125	***0.043***	0.253

**Table 6:** Rotations on the Z-axis. Changes in radial inclination presented in degrees as median (IQR). Negative values correspond to increased, while positive values to decreased radial inclination. P-values of the Mann-Whitney U test for the two groups is also presented

Group/ Time	6 weeks	3 months	6 months	1 year
Control	0.02 (0.81)	0.11 (1.01)	0.16 (1.41)	0.06 (0.94)
Hydroset	0.15 (0.69)	0.08 (1.24)	0.07 (0.97)	0.09 (1.20)
**P-values**	0.916	0.822	0.673	0.970

**Table 7:** Translations on the X-axis. Radial shift (ad latus translation) of the distal radius segment presented in mm as median (IQR). Negative values correspond to ulnar translation and positive values to radial translation. P-values of the Mann-Whitney U test for the two groups is also presented

Group/ Time	6 weeks	3 months	6 months	1 year
Control	0.00 (0.42)	0.04 (0.50)	0.00 (0.64)	0.01 (0.60)
Hydroset	0.08 (0.24)	0.04 (0.23)	0.04 ((0.38)	0.04 (0.53)
**P-values**	0.446	1.000	0.550	0.493

### Clinical outcomes and proms

ROM, grip strength, Quick-DASH, PRWE and VAS improved in absolute numbers in both groups at 1 year, however there were no significant differences between the two groups in any parameter at 1 year (Tables 8 and 9).

**Table 8:** ROM values for the control and Hydroset-group presented in degrees as median (IQR) preoperatively and at 1 year. P-values of the Mann-Whitney U test on ROM for the two groups are also presented

Group	Time	Pronation	Supination	Flexion	Extension	Radial deviation	Ulnar deviation
Control	Preop	60 (16)	70 (15)	43 (25)	64 (21)	13 (5)	23 (15)
	1 year	70 (8)	80 (15)	50 (25)	65 (21)	15 (6)	25 (10)
Hydroset	Preop	60 (15)	70 (15)	40 (25)	64 (10)	15 (10)	15 (5)
	1 year	70 (10)	80 (10)	58 (10)	65 (10)	16 (10)	25 (5)
P-values	Preop	0.61	0.58	0.59	0.58	0.53	0.42
	1 year	0.83	0.70	0.48	0.87	0.42	0.22

**Table 9:** Grip strength (kg), Quick-DASH, PRWE, and VAS-pain are reported as median (IQR) for the control and hydroset groups, as well as Mann-Whitney U test P-values preoperatively and at 1 year

Group	Time point	Grip strength	Quick-DASH	PRWE	VAS-pain
Control	Preop	18 (11)	36.4 (27.3)	58 (43)	42 (33)
	1 year	25.8 (10.2)	20.4 (36.5)	22.8 (34.6)	25 (38.8)
Hydroset	Preop	14.7 (24.7)	45.5 (29.5)	56.3 (42.9)	43 (49)
	1 year	29.2 (15.2)	15.9 (22.7)	14.8 (23.6)	14 (20)
P-values	Preop	0.41	0.77	0.89	0.76
	1 year	0.75	0.13	0.32	0.31

## Discussion

This study showed that, up to one year after distal radius osteotomy, the distal radius segment in osteotomies treated with HA exhibited significantly less movement in dorsal/palmar tilt at all time points and significantly less axial compression (proximal longitudinal translation) at 6 weeks and 6 months compared to the control group, where the osteotomy gap was left unfilled. ROM, grip strength, and PROMS improved in both groups over time, but there were no significant differences in any parameter between the groups at 1 year.

The introduction of palmar plates with locking screws has revolutionized the treatment of both DRFs and corrective osteotomies after DRM. An osteotomy for a DRM aims to reposition the distal segment in a more anatomical position whereafter a palmar plate is used to stabilize the proximal and distal segments. This often creates a gap dorsally, which is filled with autologous bone or IBS. Filling the gap likely increases the stability of the whole osteotomy to some extent, thus decreasing the risk for movement in the distal segment and could also decrease the risk for plate and/or screw failure. Biomechanical studies of palmar locking radius plates have shown that these plates are very rigid to bending and shearing forces [39]. Interestingly, recent studies have shown that it is possible to leave the osteotomy gap unfilled, with some studies suggesting that some palmar cortical contact is beneficial [1, 10] whereas other studies indicate that bone contact is not needed [11]. But there are no studies on how wide and long the gap can be before the bone regeneration process cannot overbridge it. Likely the plate is loaded more in cases where no material is used to fill the gap and in theory the risk for plate and/or screw failure would be higher in cases where the gap is not filled. However, even if the HA group showed significantly less dorsal/palmar tilt and less axial compression compared to the control group, it is important to point out that the absolute differences in loss of angulation and axial height respectively are in the subclinical scale [40] and the difference in micromotion between using bone substitute and not is likely not clinically significant. No differences were observed between treatments in terms of lateral translation and radial inclination within the first post-operative year. While research interest has been evident in treating malunions following distal radius fractures with various means, such as injectable bone substitutes [1, 2, 8, 15, 41–43], very few studies have focused on the impact that these procedures have on distal radius interfragmental stability as measured using marker-based RSA analysis. Mulders et al. [17] studied secondary displacement on conventional radiographs in open-wedge corrective osteotomies of the distal radius in 48 patients over time with or without the use of bone graft when no cortical contact could be preserved. In their study, autologous or allogenic bone graft from the iliac crest or demineralised bone matrix (DBM) was used, and the osteotomy was stabilized with a palmar plate. At a minimum of six months after surgery, no differences were reported in dorsal/palmar tilt, however there was a significant difference in radial inclination and axial compression (proximal longitudinal translation) in favour of the DBM. These differences were, though, minimal and seemed to lack clinical importance, as no differences were noted in PROMs (DASH and PRWE), reoperations due to implant failure or time to union. Although, both bone grafts and DBM were used in that study when no cortical contact was present and the radiological evaluation was done on conventional radiographs, the current study corroborates the findings by Mulders et al. [17] showing that bone substitute does not seem to provide any additional clinical advantage, in terms of inter-fragmental stability in the distal radius after a corrective open wedge osteotomy. Hydroxyapatite IBS harden through crystallization within minutes after injection, achieving initial compressive strength of 5–10 MPa. They reach near-maximum strength in 4–8 h and a final strength of 20–60 MPa within 24 h, comparable to or exceeding cancellous bone strength [44]. However, full structural stability takes 6–12 months, as their porosity and crystalline structure promote osteoconductive and osteointegrative processes, remodelling into bone via osteoclastic resorption and osteoblastic activity [13]. In contrast, cortico-cancellous bone grafts, the gold standard for filling osteotomy gaps, achieve union faster (6–12 weeks) and could theoretically provide structural support directly. IBS, such as HydroSet, while effective in providing a scaffold, rely on the body’s native healing, leading to union times similar to or longer than cortico-cancellous grafts (up to 6–12 months). Interestingly, recent studies, among them a recent review of 12 studies on extraarticular distal radius osteotomies questioned the necessity of filling the osteotomy gap altogether. They found that osteotomies stabilized with a palmar locking plate, where some palmar cortical contact was maintained, achieved union and functional outcomes comparable to those using grafts [2, 43]. Additionally, a more recent study also questioned the need to fill the osteotomy gap even in cases with no volar cortical contact and significant relevant lengthening of the radius, showing complete healing and no secondary loss of reduction, except one patient [11].

Previous studies have indicated that corrective osteotomy of the distal radius, both with and without the use of injectable bone substitutes (IBS), is associated with improved clinical outcomes in terms of function, disability, and pain [15, 45]. However, Mulders et al. [17], reported no significant differences in PROMs between patients treated with or without bone graft or demineralized bone matrix (DBM), though their study did not include the use of IBS and evaluated only two PROMs (DASH and PRWE). Similarly, Andreasson et al. [46], found no differences in clinical outcomes, including grip strength, range of motion (ROM), and PROMs, between patients who underwent corrective osteotomy for DRM with or without IBS, though they did not correlate these findings with radiographic outcomes. To our knowledge, no studies have specifically examined micromotions using radiostereometric analysis (RSA) alongside clinical outcomes and PROMs in patients with IBS-filled versus unfilled osteotomy gaps following distal radius osteotomy. In the current study, clinical outcomes and PROMs (grip strength, ROM, Quick-DASH, PRWE, and VAS-pain) were similar between the two groups, suggesting that IBS does not provide additional benefit for patient function or pain.

Two patients were excluded from the analysis due to failure of the palmar plate after a traumatic fall between 6 and 12 weeks postoperatively before the osteotomy was healed. Both patients belonged to the control group, but since it was only two patients and the fact that significant trauma was involved, it is difficult to draw any conclusions about the rigidness, stability, and healing times of osteotomies without injectable bone substitute. However, previous research on open-wedge osteotomies of the distal radius, showed that the risk of reoperation due to implant failure was similar with or without the use of bone graft or bone substitute [17]. The plate used in this study was radiolucent to facilitate the RSA analysis and made of PEEK that is inherently not as rigid as modern titanium locking plates, but to which extend this fact affects stability of osteotomies for DRM when PEEK plates are used, remains unclear [47]. If one is to leave the osteotomy gap after a distal radius osteotomy unfilled, then it is important to use stable implants while maintaining some palmar cortical contact if possible.

Although our study focuses on micromotions and the use of IBS, the role of preoperative 3D planning software is highly relevant for ensuring that the osteotomy is executed as planned. This precision could theoretically impact the initial stability of the construct by optimizing plate and screw placement [48] and could therefore indirectly influence the observed micromotions, highlighting the importance of incorporating such technology into clinical practice.

This study has limitations, the number of participants was small, which may impact the study’s generalizability. Future research should focus on prospective randomized studies to further investigate if corrective osteotomies of the distal radius without graft heal even when palmar bone contact is not preserved or if the use of hydroxyapatite bone substitute in cases where no cortical contact can be maintained can promote faster and more stable healing. Further studies should also assess if there is a limit for how big gap can be left without grafting or filling with IBS. Moreover, future studies might also focus on a clear comparison between IBS and corticocancellous or cancellous bone grafts in terms of stability and healing time. An additional limitation was that, due to a problem with the randomization software there was a minor imbalance between the groups, 25 vs 27 instead of 26 participants in each group but the size of the groups was considered large enough to complete the study. Osteotomy of malunited distal radius fractures can be performed through a palmar, dorsal or combined palmar/dorsal approach with the most common being the palmar. However, at our department osteotomies are done either using a palmar approach only or a combined approach. For the purpose of this study, we deemed it was advantageous to use a combined approach and wedges as a reposition aid (see ’’Surgical Technique’’), because it allowed perfect control of the osteotomy gap to secure that some palmar bone contact remains and that in cases where IBS was used that the filling was complete. It is acknowledged that dual approaches could increase the risk of soft tissue trauma. However, meticulous surgical technique, including careful dissection and soft tissue handling, was emphasized to mitigate these risks. Additionally, patients in this study underwent structured postoperative rehabilitation to manage oedema and reduce the risk of CRPS and no cases of postoperative CRPS were noted in this cohort. Despite these precautions, future studies comparing single and dual-approach techniques in terms of soft tissue outcomes and overall complication rates would be valuable in refining surgical strategies.

## Conclusion

The use of a hydroxyapatite bone substitute in the osteotomy gap offers no additional clinical benefit in extra-articular corrective osteotomy for malunited distal radius fractures when fixation is achieved with a palmar plate and some palmar bone contact is maintained.

## Data Availability

No datasets were generated or analysed during the current study.
